# Antibody-based PET imaging of amyloid beta in mouse models of Alzheimer's disease

**DOI:** 10.1038/ncomms10759

**Published:** 2016-02-19

**Authors:** Dag Sehlin, Xiaotian T. Fang, Linda Cato, Gunnar Antoni, Lars Lannfelt, Stina Syvänen

**Affiliations:** 1 Department of Public Health and Caring Sciences/Geriatrics, Uppsala University, Rudbeck Laboratory, 75185 Uppsala, Sweden; 2 Department of Medicinal Chemistry, Preclinical PET Platform, Uppsala University, 75123 Uppsala, Sweden; 3 PET Centre, Uppsala University Hospital, 75185 Uppsala, Sweden

## Abstract

Owing to their specificity and high-affinity binding, monoclonal antibodies have potential as positron emission tomography (PET) radioligands and are currently used to image various targets in peripheral organs. However, in the central nervous system, antibody uptake is limited by the blood–brain barrier (BBB). Here we present a PET ligand to be used for diagnosis and evaluation of treatment effects in Alzheimer's disease. The amyloid β (Aβ) antibody mAb158 is radiolabelled and conjugated to a transferrin receptor antibody to enable receptor-mediated transcytosis across the BBB. PET imaging of two different mouse models with Aβ pathology clearly visualize Aβ in the brain. The PET signal increases with age and correlates closely with brain Aβ levels. Thus, we demonstrate that antibody-based PET ligands can be successfully used for brain imaging.

## Abstract (web summary)

Imaging tools for evaluating progression of Alzheimer's disease have been lacking. Here the authors develop a blood brain barrier-permeable Aß probe based on a radiolabelled, anti-Aß antibody, and report age-dependent brain uptake visualized in vivo with PET in mouse models of the disease.

## Graphical abstract

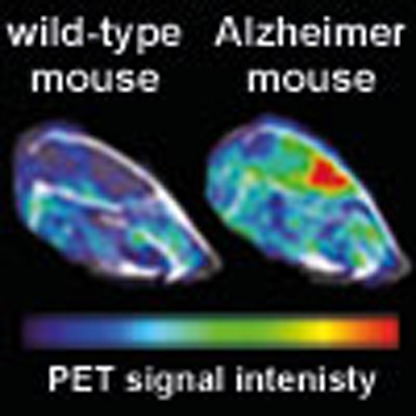

Positron emission tomography (PET) imaging of amyloid β (Aβ) deposits in the brain has rapidly advanced in recent years. The introduction of the radioligand [^11^C]PIB (ref. 1), a derivate of thioflavin-T, was an important development for diagnosis of Alzheimer's disease (AD), as [^11^C]PIB amyloid imaging detects AD pathology early in the course of disease^2^ and helps distinguishing AD from other types of dementia^3^,^4^. [^11^C]PIB, and analogues of PIB, detect amyloid plaques, mainly consisting of insoluble fibrils of Aβ (ref. 1). However, the load of insoluble Aβ does not correlate well with disease progression^5^,^6^. Soluble Aβ is a better marker of disease status^7^,^8^,^9^,^10^, and many therapeutic as well as diagnostic efforts are currently targeting soluble Aβ aggregates, for example, oligomers and protofibrils^11^,^12^,^13^,^14^,^15^,^16^,^17^, which are strongly implicated as the cause of synaptic failure and neurodegeneration in AD^9^,^10^,^18^,^19^,^20^,^21^,^22^,^23^. This new focus highlights the pressing need for an imaging agent that can visualize soluble Aβ aggregates. The development of small molecular PET radioligands often suffers from nonspecific binding of the radioligand, and further, low ability to discriminate between different forms of a protein. Radioligands based on antibodies have recently been introduced in clinical use for various peripheral antigens primarily related to cancer^24^. Antibodies have the advantage that they can be developed to bind a specific form of a protein, but their use as PET radioligands for targets in the central nervous system (CNS) is hampered by their low brain penetration. However, whereas a high enough brain uptake is essential to achieve a PET signal, the specific-to-nonspecific binding, expected to be very high for a monoclonal antibody compared with small molecules, may be equally important.

Our previously developed conformation-selective monoclonal antibody mAb158 displays a distinctive selectivity for soluble Aβ protofibrils as compared with monomeric Aβ. It binds preferentially to soluble protofibrils over mature, insoluble fibrils, but without affinity for the Aβ protein precursor (AβPP)^25^,^26^,^27^,^28^. These characteristics make the antibody suitable to selectively target soluble Aβ aggregates *in vivo*, and its humanized version, BAN2401, (hereafter referred to as h158), is currently studied in a phase 2b clinical trial as an anti-Aβ therapy against AD. In a previous study we showed that 72 h after administration, the brain concentrations of iodine-125-labelled mAb158 ([^125^I]mAb158) was significantly increased in a transgenic AD mouse model (tg-ArcSwe, harbouring the Arctic (*E693G*) and Swedish (*KM670/671NL*) AβPP mutations^29^) compared with non-transgenic littermates (wild type; WT)^27^. However, total levels of [^125^I]mAb158 in the brain were rather moderate, which is anticipated for antibodies in general^30^.

Different strategies have been applied to increase the brain uptake of antibodies and other large molecules. Among them is receptor-mediated transcytosis, where the antibody is fused to a molecule that specifically binds to a blood–brain barrier (BBB) expressed receptor, for example, the transferrin or insulin receptor, which enables active transport across the BBB (Fig. 1a). This technique was pioneered by Pardridge and colleagues^31^ in the 1990s, and has recently been implemented in AD therapy to decrease production^32^,^33^ or increase degradation^34^ of Aβ.

In the present study, a F(ab′)_2_ fragment of h158 is chemically fused to a transferrin receptor (TfR) antibody^35^ with the aim to create a PET ligand for specific imaging of soluble Aβ protofibrils. Brain retention of the generated bispecific fusion protein increases 15-fold, compared with F(ab′)_2_-h158. We then show by PET imaging that the brain distribution of ^124^I-labelled fusion protein correlates closely with the age-dependent increase of Aβ pathology in the brains of two transgenic mouse models with AD-like pathology. This new radioligand has the potential to become an important diagnostic tool in AD and furthermore, the study demonstrates that bispecific radioligands based on antibodies can be applied in medical imaging of proteins associated with CNS disorders.

## Results

### Engineering of an Aβ–TfR bispecific fusion protein

A PET ligand with a fairly short systemic half-life is desired since a rapid elimination from the blood increases the specific signal compared with the background, derived from the blood volume of the studied tissue (about 5% in brain) and decreases the radiation dose for the patient. Therefore, a F(ab′)_2_ fragment was generated by enzymatic cleavage of h158, reducing its systemic half-life to ∼2 h in both tg-ArcSwe and WT mice, compared with 11 days for mAb158 (ref. 27). The Aβ-binding properties of F(ab′)_2_-h158 were unchanged, compared with the parent antibody. To increase brain uptake of F(ab′)_2_-h158, it was chemically conjugated to the anti-TfR antibody 8D3 (ref. 36), which has been widely used to increase the brain uptake of large molecules^34^,^37^,^38^,^39^,^40^.

**Figure 1:**
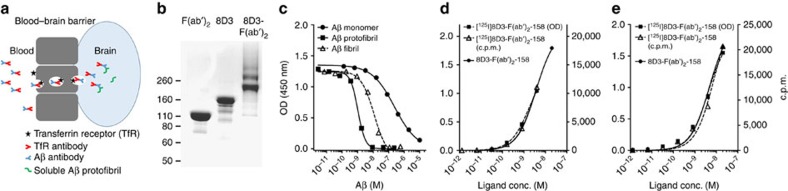
Generation of bispecific fusion protein 8D3-F(ab′)_2_-h158. (**a**) Schematic picture of TfR-mediated transcytosis. (**b**) SDS–PAGE displaying F(ab′)_2_-h158, TfR antibody 8D3 and the fusion protein consisting of F(ab′)_2_-h158 and 8D3. (**c**) Inhibition ELISA demonstrating the fusion protein's selective binding to Aβ protofibrils (IC_50_ 1.0 nM) over fibrils (IC_50_ 12 nM) and monomers (IC_50_ 240 nM). The fusion protein showed retained binding to TfR (**d**) and Aβ protofibrils (**e**) after ^125^I labelling, as demonstrated with ELISA (absorbance values on the left *y* axis and radioactivity on the right *y* axis). Representative images from triplicate experiments are shown in **c**–**e**. conc., concentration; c.p.m., counts per minute; IC_50_, median inhibitory concentration; OD, optical density; SDS–PAGE, SDS–polyacrylamide gel electrophoresis.

The 8D3-F(ab′)_2_-h158 fusion protein was sufficiently pure for the intended purpose (Fig. 1b). The difference in fusion protein binding in solution to Aβ monomer, protofibril and fibril, reflected by their ability to inhibit the fusion protein's binding to an Aβ-coated enzyme-linked immunosorbent assay (ELISA) plate, was very similar to what we have previously reported for mAb158 (refs 25, 27), with median inhibitory concentration values of 240 nM for monomers, 1.0 nM for protofibrils and 12 nM for fibrils (Fig. 1c). Furthermore, it retained its binding to both TfR and Aβ protofibrils also after radioiodination (Fig. 1d,e).

### Increased brain uptake by TfR-mediated transcytosis

**Figure 2:**
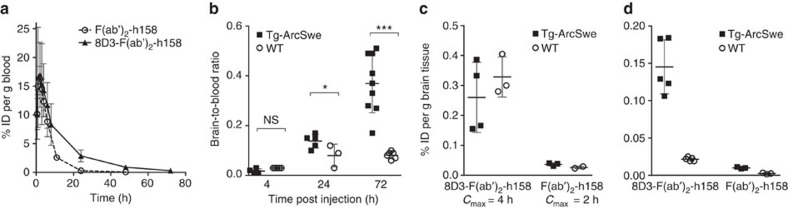
*In vivo* blood pharmacokinetics and brain distribution of fusion protein in tg-ArcSwe and WT mice. (**a**) The bispecific fusion protein (triangles, *n*=6) showed an increased half-life (11 h) compared with unmodified F(ab′)_2_-h158 (circles, *n*=3; 2 h). (**b**) The brain-to-blood concentration ratio of the fusion protein increased over time in tg-ArcSwe mice (>12 months, *n*=18) while remaining fairly constant in WT mice (*n*=14). (**c**) Comparison of brain distribution of fusion protein (*n*=7) and unmodified F(ab′)_2_-h158 (*n*=5) at respective *C*_max_. At this time point the increase in brain distribution mainly reflected increased transport across the BBB as the increase was observed both in tg-ArcSwe and WT mice (>18 months). (**d**) Comparison of brain distribution of fusion protein (*n*=10) and unmodified F(ab′)_2_-h158 (*n*=6) 72 h post injection. At this time point the differences observed between tg-ArcSwe and WT mice (>18 months) reflected binding to Aβ protofibrils. The symbols and error bars indicate group mean±s.d. from experiments (**a**). Each symbol represents one animal, line and error bars indicate group mean±s.d. (**b**–**d**). *P<0.05, ***P<0.001 and NS is nonsignificant by two-way analysis of variance followed by Bonferroni's *post hoc* test (**b**).

The pharmacokinetic blood profile of the radioiodinated fusion protein [^125^I]8D3-F(ab′)_2_-h158 was the same in tg-ArcSwe and WT mice displaying a half-life of 11 h (Fig. 2a). For successful PET imaging, it is important to have a high brain uptake of the ligand in relation to its concentration in blood. Therefore, the brain-to-blood ratio (*K*_p_) of the fusion protein was assessed by *ex vivo* studies at three different time points. Radioactivity was measured in blood and saline-perfused brains of >12-month-old tg-ArcSwe and WT mice, killed 4, 24 and 72 h after intraperitoneal (i.p.) injection of [^125^I]8D3-F(ab′)_2_-h158. At 4 h *K*_p_ was low (0.02±0.01), with no difference between tg-ArcSwe and WT mice. *K*_p_ had increased at 24 h and was significantly different (*P*<0.05) between the groups. At 72 h post injection, *K*_p_ had further increased to 0.44±0.10 (*P*<0.001) in tg-ArcSwe, while the WT mice were stable on a low level (Fig. 2b). The fusion protein transport into the brain was assessed in >18-month-old mice at 4 and 2 h, corresponding to *C*_max_ of the fusion protein and the F(ab′)_2_ fragment in blood, respectively, and measured as per cent of injected dose per gram brain tissue (% ID per g). At this point, the fusion protein was taken up ninefold more than [^125^I]F(ab′)_2_-h158, with no difference between tg-ArcSwe and WT mice (Fig. 2c). When studied 72 h post injection, that is, at the highest *K*_p_ of the fusion protein, it showed a 15-fold higher brain retention in tg-ArcSwe mice than the F(ab′)_2_ fragment and a greater difference between tg-ArcSwe and WT mice (Fig. 2d).

**Figure 3:**
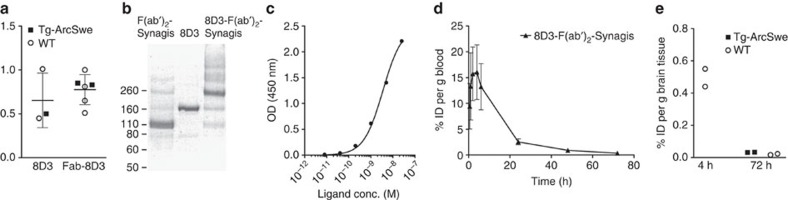
Brain distribution of 8D3 and generation of an Aβ irrelevant fusion protein. (**a**) Brain distribution of 8D3 (*n*=3) and Fab-8D3 (*n*=6) in 10- to 12-month-old WT and tg-ArcSwe mice 4 h post injection, demonstrating that the TfR-mediated transcytosis of 8D3 is Fc-independent. (**b**) SDS–PAGE displaying F(ab′)_2_-Synagis, TfR antibody 8D3 and the fusion protein consisting of F(ab′)_2_-Synagis and 8D3. (**c**) TfR ELISA demonstrating that the irrelevant fusion protein binds to TfR *in vitro*. (**d**) *In vivo* blood pharmacokinetics of irrelevant fusion protein in tg-ArcSwe (*n*=2) and WT mice (*n*=4): the irrelevant fusion protein showed a similar half-life as the 8D3-F(ab′)_2_-h158 fusion protein (11 h). (**e**) Comparison of irrelevant fusion brain concentration at 4 h (*C*_max_), demonstrating an increased transport across the BBB in WT mice (*n*=2), and at 72 h post injection, where the low concentration of irrelevant fusion protein in both tg-ArcSwe (*n*=2) and WT (*n*=2) mice reflects the lack of target in the brain. The symbols and error bars indicate group mean±s.d. (**d**). Each symbol represents one animal (**a** and **e**). SDS–PAGE, SDS–polyacrylamide gel electrophoresis.

To assess whether the transport across the BBB may be mediated by the Fc receptor, 10- to 12-month-old WT and tg-ArcSwe mice were injected with ^125^I-labelled 8D3 or a Fab fragment of 8D3 (Fab-8D3), which lacks the Fc fragment, and *ex vivo* brain uptake was measured 4 h post injection. Fab-8D3 was equally well distributed to the brain as 8D3, strongly indicating that the high brain concentrations observed with 8D3 and the fusion protein were due to TfR-mediated transcytosis and independent of the Fc domain of the 8D3 antibody (Fig. 3a). As an additional control, a fusion protein consisting of 8D3 and a F(ab′)_2_ fragment of an antibody (Synagis; specific for the respiratory syncytial virus) of the same IgG isoform as h158, but lacking a specific target in the brain, was also generated. The generated 8D3-F(ab′)_2_-Synagis fusion protein retained its binding to TfR, but did not bind to Aβ protofibrils (Fig. 3b,c).

*Ex vivo* studies were also performed with the irrelevant fusion protein [^125^I]8D3-F(ab′)_2_-Synagis, which had a similar half-life in blood as [^125^I]8D3-F(ab′)_2_-h158. Similar to 8D3, the brain retention of the irrelevant fusion protein was elevated in WT mice 4 h post injection. At 72 h post injection, the brain retention of the irrelevant fusion protein was the same in tg-ArcSwe and WT mice (Fig. 3d,e) and of the same magnitude as observed with [^125^I]8D3-F(ab′)_2_-h158 in WT mice.

These experiments demonstrated that compared with F(ab′)_2_-h158, the fusion protein transport into the brain was markedly increased in both tg-ArcSwe and WT mice. Hence, the carrier-mediated transcytosis was not dependent on animal type or age. However, in WT animals, lacking Aβ protofibrils, the fusion protein was washed out from the brain when systemic concentrations decreased. Similarly, [^125^I]8D3-F(ab′)_2_-Synagis, which lacks a protofibril-binding domain, was eliminated from the brains of both WT and tg-ArcSwe mice when the blood concentration decreased. As a consequence of the fairly rapid systemic elimination of the fusion protein, *K*_p_ increased in tg-ArcSwe mice over time, but not in WT mice. Moreover, when compared with the whole antibody mAb158 (ref. 27), *K*_p_ of the fusion protein was more than 20-fold increased 72 h post injection.

### Brain retention follows Aβ pathology in transgenic mice

**Figure 4:**
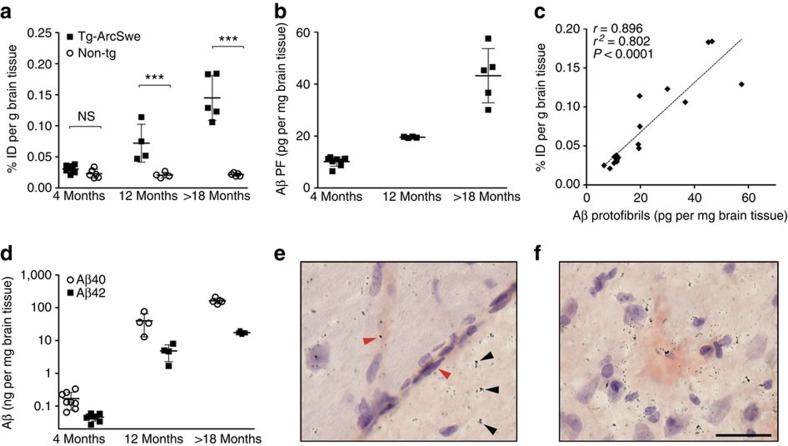
Age-dependent retention of fusion protein in brain. (**a**) *Ex vivo* brain retention expressed as % ID per g brain tissue in tg-ArcSwe (*n*=17) and WT (*n*=15) mice of different ages 72 h post injection. (**b**) Aβ protofibril levels in brain tissue obtained from tg-ArcSwe mice of different ages. (**c**) Pearson's correlation analysis of brain concentrations of fusion protein 72 h post injection and concentration of Aβ protofibrils in brain tissue of tg-ArcSwe mice. Each diamond represents one animal. (**d**) Total Aβ40 and Aβ42 levels in brain tissue from tg-ArcSwe mice of different ages. (**e**) Nuclear track emulsion and CD31 staining. The fusion protein was not accumulated in capillaries (red arrows) but to a large extent reached the brain parenchyma (black arrows). (**f**) Nuclear track emulsion and Congo staining revealed that the fusion protein was also located around insoluble amyloid deposits. Scale bar, 50 μm. Each symbol represents one animal, the line and error bars (**a**–**d**) indicate group mean±s.d. ***P<0.001 and NS is nonsignificant by two-way analysis of variance followed by Bonferroni's *post hoc* test (**a**). Representative images from triplicate experiments are shown in **e** and **f**.

The brain retention of the fusion protein was measured *ex vivo* 72 h post injection in saline-perfused brains of tg-ArcSwe and WT mice at 4, 12 and >18 months of age to follow the course of Aβ pathology. Brain levels of soluble Aβ protofibrils as well as total (formic acid soluble) Aβ40 and Aβ42 were determined in the same mice. We have previously established that total Aβ, measured by ELISA, closely matches Aβ plaque load as determined with immunohistochemistry in tg-ArcSwe mice^27^. There was a significant difference in brain retention of the fusion protein between tg-ArcSwe and WT mice at 12 months (3.5-fold) and >18 months (6.8-fold), as well as a trend towards increased brain concentrations also in the 4-month group (1.3-fold) (Fig. 4a and Table 1). The WT animals showed constant brain concentrations of fusion protein regardless of age. Both soluble Aβ protofibrils and total Aβ40 and Aβ42 increased over time in tg-ArcSwe mice, displaying elevated levels of Aβ protofibrils already at 4 months (Fig. 4b). The increase over time in soluble Aβ protofibril levels correlated closely with the brain concentrations of fusion protein (Fig. 4c), while total Aβ concentrations increased with a higher rate (Fig. 4d) suggesting that the Aβ targeted by the fusion protein was mainly of soluble origin. To rule out the possibility that the ligand might be stuck in the endothelium and not transported into the brain, staining for the endothelial marker CD31 in combination with nuclear track emulsion was used to visualize the fusion protein in the brain. The fusion protein was found in the brain parenchyma without accumulation in capillaries in >18-month-old tg-ArcSwe mice (Fig. 4e) but to some extent clustered around the periphery of congophilic amyloid deposits, suggesting it was bound to a halo of soluble Aβ aggregates, which has been reported to surround the fibrillar core of amyloid plaques^41^ (Fig. 4f).

### *In vivo* PET imaging of Aβ pathology in transgenic mice

**Figure 5:**
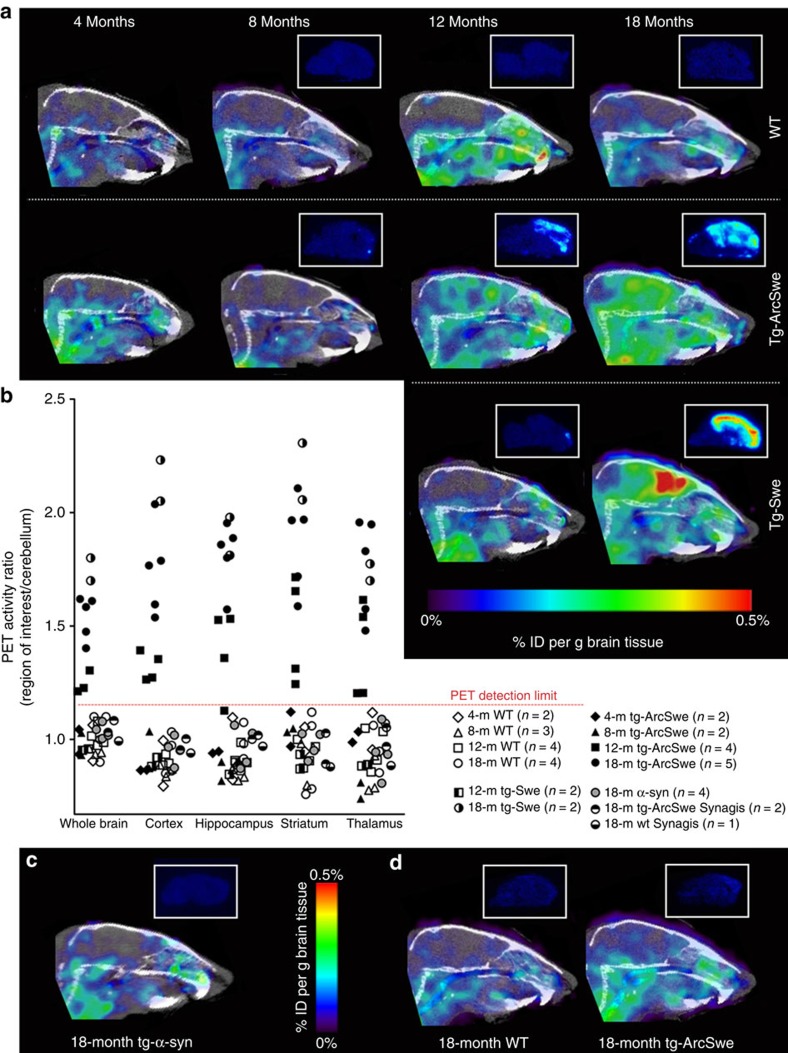
Fusion protein PET imaging in transgenic and WT mice. (**a**) Comparison of PET images obtained during 60 min, 72 h post injection of [^124^I]8D3-F(ab′)_2_-h158 from representative WT, tg-ArcSwe and tg-Swe mice of different ages, demonstrating the progression of Aβ pathology. *Ex vivo* autoradiography brain images from the same animals are displayed above PET images for comparison of brain distribution. (**b**) PET image-based quantification of brain distribution of the fusion protein relative to that in cerebellum for WT, tg-ArcSwe, tg-Swe and tg-α-syn mice in the different age groups (each symbol represents one animal). (**c**) PET image of 18-month-old tg-α-syn mouse, 72 h post injection of [^124^I]8D3-F(ab′)_2_-h158. (**d**) PET images obtained with the irrelevant [^124^I]8D3-F(ab′)_2_-Synagis 72 h post injection, with *ex vivo* autoradiography brain images from the same animals displayed above, demonstrating low and equal brain uptake in 18-month-old WT and tg-ArcSwe mice. Representative PET images are shown in **a**,**c** and **d**. Number of animals included in each group is shown in **b**.

Next, to evaluate the fusion protein as a PET ligand, it was labelled with ^124^I, a positron-emitting radionuclide with a half-life of 4.2 days. Tg-ArcSwe and WT mice of different ages (4, 8, 12 and 18 months) as well as tg-Swe mice (12 and 18 months), with a delayed age at onset of pathology compared with tg-ArcSwe^29^,^42^, were injected i.p. with ∼15 MBq [^124^I]8D3-F(ab′)_2_-h158 and PET scanned for 60 min 72 h post injection. Aβ pathology could be clearly visualized in the 12- and 18-month-old tg-ArcSwe mice while there was no signal in the WT mice (Fig. 5a). In tg-Swe mice, no specific signal was recorded at 12 months, whereas a strong signal was seen at 18 months, confirming that the signal seen in tg-ArcSwe mice was not an effect of the Arctic AβPP mutation. In a subset of the animals, *ex vivo* autoradiography was performed, as a comparison to the results of brain distribution seen in the PET images. Clinical PET data, including studies using [^11^C]PIB, is often quantified as the relative concentration of the PET ligand in a region of interest to that of a reference region. When analysing the present PET data from the whole brain, cortex, hippocampus, striatum and thalamus using the cerebellum as a reference region, the obtained ratio followed the disease progression in all studied regions (Fig. 5b). The cerebellum displayed a low PET signal in all animals at all ages (Fig. 5a) confirming its suitability as a reference region. Deiodination of the fusion protein, measured in plasma after PET scanning, was <5%.

To verify that neurodegenerative pathology characterized by protein aggregation *per se* did not lead to increased retention of [^124^I]8D3-F(ab′)_2_-h158, 18-month-old (Thy-1)-h[A30P]α-synuclein transgenic mice (hereafter referred to as tg-α-syn) were also PET scanned according to the same protocol. The tg-α-syn mice, like the WT mice, displayed no signal in the brain (Fig. 5c). In addition, to confirm that the differences in brain retention observed between AD and WT mice were not due to increased binding of the 8D3 moiety in the brains of tg-ArcSwe and tg-Swe mice, 18-month-old tg-ArcSwe and WT mice were injected with ∼15 MBq of the irrelevant fusion protein [^124^I]8D3-F(ab′)_2_-Synagis and PET scanned for 60 min 72 h post injection. No accumulation in brain could be observed in either mouse type (Fig. 5d), and when quantified, the concentration ratios in all studied brain regions in relation to cerebellum were around 1 (Fig. 5b), confirming that the higher brain concentrations observed with [^124^I]8D3-F(ab′)_2_-h158 in tg-ArcSwe and tg-Swe compared with WT mice were indeed due to the Aβ protofibril-binding moiety F(ab′)_2_-h158.

### PET imaging correlates with soluble Aβ protofibrils

**Figure 6:**
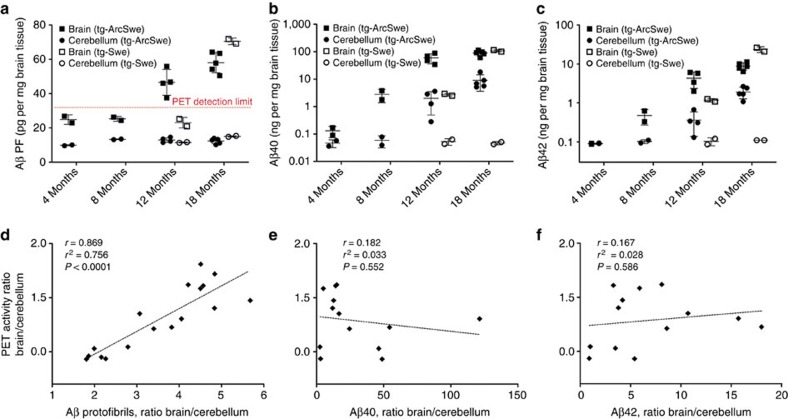
Aβ levels in brain and cerebellum and correlation with PET data. (**a**) Aβ protofibril (PF) levels in brain tissue obtained from tg-ArcSwe (*n*=13) and tg-Swe (*n*=4) mice of different ages; total Aβ40 (**b**) and Aβ42 (**c**) levels in brain and cerebellum from tg-ArcSwe and tg-Swe mice of different ages; Pearson's correlation analysis of brain/cerebellum PET ratio and brain/cerebellum concentration ratio of soluble Aβ protofibrils in tg-ArcSwe and tg-Swe mice (**d**) and of total Aβ40 (**e**) and Aβ42 (**f**) in tg-ArcSwe mice. Each symbol represents one animal previously subjected to PET scanning; the line and error bars (**a**–**c**) indicate group mean±s.d.

Brains from the mice subjected to PET imaging were analysed for Tris-buffered saline (TBS)-soluble Aβ protofibrils and total (formic acid-soluble) Aβ40 and Aβ42. Cerebellum and the rest of the brain were analysed separately to enable a direct comparison with PET results. As displayed in Fig. 6a, cerebellum contained overall very low levels of Aβ protofibrils, but more importantly, no increase was seen with increased age in either tg-ArcSwe or tg-Swe mice in direct analogy with PET results. In the rest of the brain, Aβ protofibril levels increased with age after 8 months in tg-ArcSwe, showing elevated levels at 12 months, that is, at the same age as Aβ was detectable with PET, that were further increased at 18 months. The same pattern was observed for the tg-Swe animals, although the increase was seen between 12 and 18 months. Hence, there was no overlap in Aβ protofibril levels between PET-positive and PET-negative animals, which allows to define a PET detection limit completely separating the groups (Fig. 6a). For total Aβ40 and Aβ42, the pattern was different in tg-ArcSwe mice, with a marked increase in brain Aβ concentrations already at 8 months, continuing up to 12 months and then forming a plateau. Interestingly, unlike soluble Aβ protofibril levels, total Aβ40 and Aβ42 levels increased with age also in the cerebellum, though with several months delay (Fig. 6b,c). The tg-Swe model showed a large increase in total Aβ at 18 months compared with 12 months, but low levels in the cerebellum at both ages, possibly because of its later onset of Aβ pathology (Fig. 6b,c). For comparison with the PET data, a brain/cerebellum ratio of the three different Aβ species was plotted against the brain/cerebellum PET ratio described above (Fig. 6d–f). The tg-Swe animals were excluded from the Aβ40 and Aβ42 ratio graphs since they had extremely high ratios, caused by the low Aβ levels in cerebellum. Whereas a strong correlation was seen between the Aβ protofibril and PET ratios, the total Aβ ratios did not correlate with PET data.

### Comparison with [^11^C]PIB

**Figure 7:**
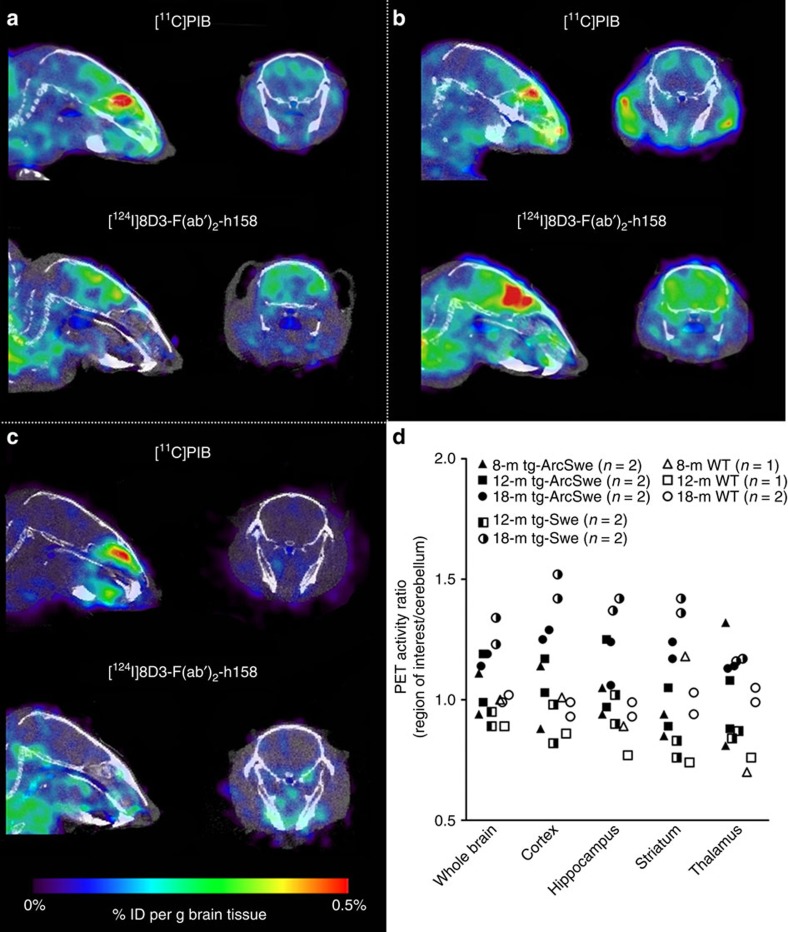
Comparison of PET imaging with [^11^C]PIB- and ^124^I-labelled fusion protein in transgenic and WT mice. PET images obtained during 60 min 72 h after injection of [^124^I]8D3-F(ab′)_2_-h158, or during 20 min, starting 40 min after injection of [^11^C]PIB. Transverse and sagittal views of one representative 18-month-old tg-ArcSwe (**a**), tg-Swe (**b**) and WT (**c**) mouse with the two radioligands. (**d**) PET image-based quantification of brain distribution of [^11^C]PIB relative to that in cerebellum for tg-ArcSwe, tg-Swe and WT mice in the different age groups (each symbol represents one animal). Representative PET images are shown in **a**–**c**. Number of animals included in each group is shown in **d**.

A subset of the mice that underwent PET scanning with the fusion protein was also imaged with [^11^C]PIB to compare the two ligands in mice with different Aβ pathology (Fig. 7). The 8- and 12-month-old transgenic animals showed similar [^11^C]PIB retention in brain as WT animals. The 18-month-old tg-ArcSwe and tg-Swe mice showed some [^11^C]PIB retention, mainly in cortical regions, but the brain/cerebellum ratio was in general lower than the ratio obtained with the fusion protein and did not reflect the disease progression to the same extent as the fusion protein did. Radioactivity concentration (corrected for injected dose) of [^11^C]PIB and [^124^I]8D3-F(ab′)_2_-h158 was similar in 18-month-old tg-ArcSwe brain (Fig. 7a), while tg-Swe mice showed 60% higher concentration of [^124^I]8D3-F(ab′)_2_-h158 than of [^11^C]PIB (Fig. 7b).

## Discussion

Several new drug compounds intended for treatment of AD have entered clinical phase II and III studies, but there are limited possibilities to measure their effects on the molecular level *in vivo*. All existing amyloid PET radioligands bind to the β-sheet structure of insoluble fibrillar Aβ. Evidence today points to soluble forms of aggregated Aβ being better correlated with disease severity and soluble Aβ oligomers and protofibrils are likely to cause the synaptic failure that eventually leads to dementia. The present study shows for the first time an *in vivo* PET image of Aβ pathology acquired with an antibody-based radioligand (Fig. 5). This was achieved by a combination of TfR-mediated transcytosis to increase BBB penetration and the use of a well-characterized Aβ protofibril selective antibody, which ensures that Aβ aggregates are selectively targeted. Although mAb158 binds, albeit with a lesser affinity, to Aβ fibrils and monomers *in vitro*^25^,^27^ (Fig. 1c), evidence suggests that it primarily targets soluble Aβ protofibrils *in vivo*. When administered to tg-ArcSwe mice with plaque pathology, mAb158 selectively reduced brain levels of Aβ protofibrils, without altering the plaque pathology^43^, unlike other Aβ antibodies of the same isotype^44^. This is probably because soluble Aβ protofibrils, which are already favoured by mAb158, are more accessible to the antibody when it enters the brain parenchyma than the insoluble fibrils deposited in plaques. This is also reflected in Fig. 4e,f, where *in vivo* administered fusion protein appears in the parenchyma and around the periphery of plaques, where Aβ oligomerization has been reported to occur^41^.

Further, the PET signal and brain retention of the generated bispecific fusion protein increased with age (Fig. 5), that is, with progression of Aβ pathology in the tg-ArcSwe and tg-Swe mouse models. Tg-ArcSwe mice exhibit very dense Aβ plaque pathology, similar to that of the human AD brain, with an onset around 6 months of age^42^, and an almost linear increase of soluble Aβ protofibrils with age (Figs 4 and 6). Tg-Swe mice display less dense plaques^42^ with a later onset and a more rapid increase in Aβ pathology (Fig. 6). Hence, at 12 months of age, higher concentrations of [^124^I]8D3-F(ab′)_2_-h158 was observed in the brains of tg-ArcSwe compared with tg-Swe mice, while the opposite was observed at 18 months. The difference between the two animal models was also evident in the [^11^C]PIB scans. [^11^C]PIB binds poorly to the loose unstructured plaques in tg-Swe mice brain and thus [^11^C]PIB concentrations were lower than [^124^I]8D3-F(ab′)_2_-h158 in tg-Swe mice, while the concentration of the two radioligands was similar in tg-ArcSwe brain. Brain retention of the fusion protein correlated closely with brain concentrations of soluble Aβ protofibrils in both animal models (Fig. 4), while total Aβ levels, reflecting the plaque pathology, increased at a higher rate, suggesting that the fusion protein preferably binds to soluble Aβ protofibrils. Furthermore, low radioactivity was detected with PET in the cerebellum in all age groups, while it increased with age in the rest of the brain. Similarly, while the level of soluble Aβ protofibrils increased with age in the whole brain, cerebellum displayed a low and constant level of protofibrils over time. Thus, the PET brain/cerebellum radioactivity concentration ratio, which is often used in clinical PET as a read-out measure, correlated with the brain/cerebellum ratio of soluble Aβ protofibrils (Fig. 6). Such a correlation was not seen for total Aβ, since both Aβ40 and Aβ42 increased with age in the cerebellum. These results do not *per se* exclude that the radioligand may also to some extent bind to fibrillar Aβ in the brain, possibly in the shape of diffuse deposits.

Taken together, our findings suggest that brain retention of the fusion protein measured with PET reflects the progression of Aβ pathology. This approach could become an important diagnostic tool to predict disease stage in AD patients, likely including the group of patients displaying mainly diffuse plaque pathology^45^ that are diagnosed falsely as non-AD with the available amyloid PET radioligands^46^. Treatment strategies to reduce soluble Aβ aggregates are today studied in late-phase clinical trials, but biomarkers for evaluating the effects of such treatments need to be improved. A PET radioligand visualizing soluble Aβ aggregates could bring substantial benefit in assessing emerging Aβ-reducing treatments.

To our knowledge this is the first successful study using an antibody-based PET ligand for a CNS application. Since antibodies are very specific binders, the drawback of large unspecific binding often experienced with small molecular PET ligands (including [^11^C]PIB and analogues) will most likely be avoided with antibody-based ligands. This study therefore also demonstrates the feasibility of antibody-based *in vivo* imaging of proteins involved in other neurodegenerative disorders, for example, Parkinson's disease or frontotemporal lobar degeneration, for which small molecular PET ligands are currently lacking.

## Methods

### Antibody fragmentation

F(ab′)_2_-h158 was generated by cleavage of a humanized variant, BAN2401, of the mouse monoclonal antibody mAb158, selectively binding to Aβ protofibrils, that is, soluble Aβ aggregates larger than about 100 kD, eluting in the void volume on a Size Exclusion Superdex 75 column^25^. The bacterial enzyme IdeS (ref. 47), manufactured and distributed as FabRICATOR (Genovis AB, Lund, Sweden), was used to cleave the antibody. This enzyme cleaves human IgG at a specific site just below the hinge region, producing a homogenous preparation of F(ab′)_2_ fragments. A F(ab′)_2_ fragment of an antibody against respiratory syncytial virus, Synagis (Palivizumab; 530300, Apoteket AB, Solna, Sweden/MedImmune, Gaithersburg, MD, USA), was generated according to the same method. Fab-8D3 was generated from the TfR antibody 8D3 (ref. 36) (MCA2474, AbD Serotec, Oxford, UK) by papain cleavage, according to the manufacturer's protocol (Pierce, Rockford, IL, USA).

All fragments were purified with CaptureSelect Fc (multi-species) Affinity Resin (Thermo Fisher Scientific, Stockholm, Sweden) to remove Fc fragments and non-cleaved antibody from the preparation. Purity and size of the fragments were evaluated with SDS–PAGE under non-reducing conditions. Briefly, samples were mixed with Laemmli buffer, loaded onto a Novex pre-cast 10–20% Tris-tricine polyacrylamide gel (Invitrogen, Carlsbad, CA) and run at 125 V for 90 min, followed by 3 × 15-min wash in water, and staining with Page Blue (Fermentas, Vilnius, Lithuania) according to the manufacturer's instructions.

### Generation of bispecific fusion proteins

F(ab′)_2_-h158 or F(ab′)_2_-Synagis and 8D3 were chemically conjugated with the Solulink technology (Solulink protein conjugation kit; Solulink, San Diego, CA, USA), where each of the conjugated proteins is modified with one of two linkers, which bind specifically to each other to form a permanent bond. F(ab′)_2_-h158 or F(ab′)_2_-Synagis (3.0 mg ml^−1^) was labelled with succinimidyl-4-formylbenzamide (S-4FB) using a 4.5-fold molar excess, and 8D3 (3.0 mg ml^−1^) was labelled with the complimentary succinimidyl-6-hydrazino-nicotinamide (S-HyNic) using a 6-fold molar excess. The labelled proteins were mixed in a 1.5:1 (F(ab′)_2_:8D3) molar ratio and reacted for 2 h in room temperature in the presence of 10 mM aniline, which catalyses the reaction. To purify the fusion protein, the preparation was incubated for 1 h with CaptureSelect Fc (multi-species) Affinity Matrix to specifically deplete the preparation of unconjugated F(ab′)_2_ fragments, which lack the Fc domain. After elution from the resin with 0.1 M glycine-HCl, pH 2.5, the preparation was neutralized with 1 M Tris and incubated for 1 h with CaptureSelect IgGCH1 (human) Affinity Matrix (Thermo Fisher Scientific), which specifically binds to the constant domain 1 of human IgG heavy chain, thus depleting the sample of unconjugated 8D3. The purified fusion protein was eluted with 0.1 M glycine-HCl, pH 2.5, and analysed with SDS–PAGE as above.

### Aβ inhibition ELISA

To assess the 8D3-F(ab′)_2_-h158 fusion protein's binding to different Aβ species in solution, an inhibition ELISA was performed as previously described^25^. The 96-well plates (Corning Inc., Corning, NY, USA) were coated at +4 °C for 2 h, with 45 ng per well of Aβ protofibrils and blocked for 1 h with 1% bovine serum albumin (BSA) in PBS. In the meantime, the fusion protein (0.5 nM) was incubated with serially diluted Aβ monomers, protofibrils or fibrils in a non-binding 96-well plate (Greiner, Kremsmünster, Austria) for 1 h on a shaker and then transferred to the Aβ protofibril-coated plate, where it was incubated for 15 min. Fusion protein bound to the plate was detected by a 1-h incubation with 1:2,000 diluted horseradish peroxidase (HRP)-conjugated anti-human-IgG-F(ab′)_2_ (109-036-006, Jackson ImmunoResearch Laboratories, West Grove, PA, USA). Signals were developed with K blue aqueous TMB substrate (Neogen Corp., Lexington, KY, USA) and read with a spectrophotometer at 450 nm. All Aβ and antibody dilutions were made in ELISA incubation buffer (PBS with 0.1% BSA, 0.05% Tween and 0.15% Kathon). Aβ preparations were made as previously described^27^ and the (insoluble) fibril preparation was subjected to a brief sonication (30 s) before the analysis to break-up large fibril assemblies and ensure it would stay in suspension.

### TfR–Aβ protofibril ELISA

The generated 8D3-F(ab′)_2_-h158 fusion protein was tested with ELISA for retained binding to TfR and Aβ, respectively, before and after radioiodination. The 96-well plates (Corning Inc.) were coated at +4 °C overnight with 10 ng per well of murine TfR (Sinobiological, Beijing, China) or 25 ng per well of streptavidin (Sigma, St. Louis, MO, USA). After 1 h blocking with 1% BSA in PBS, biotinylated Aβ protofibrils, 4.5 ng per well, prepared as previously described^27^, were added to streptavidin-coated wells and incubated for 30 min on a shaker. A serial dilution of the fusion protein, ^125^I-labelled or non-labelled, was added to TfR- or streptavidin/Aβ protofibril-coated wells, incubated for 2 h on a shaker and detected by a 1-h incubation with HRP-conjugated 1:2,000 diluted anti-mouse-IgG-F(ab′)_2_ (TfR coat) or anti-human-IgG-F(ab′)_2_ (streptavidin/Aβ protofibril coat) (115-036-006 and 109-036-006, Jackson ImmunoResearch Laboratories). Signals were developed and read as above. All antibody dilutions were made in ELISA incubation buffer. The 8D3-F(ab′)_2_-Synagis fusion protein was tested for retained binding to TfR according to the same method.

### Animals

Three transgenic models maintained on a C57BL/6 background were used: the tg-ArcSwe model harbouring the Arctic (*AβPP E693G*) and Swedish (*AβPP KM670/671NL*) mutations; the tg-Swe model with only the Swedish mutation; and tg-α-syn mice. Tg-ArcSwe mice show elevated levels of soluble Aβ protofibrils already at a very young age and abundant and rapidly developing plaque pathology starting at around 6 months of age^29^,^42^,^48^. Tg-Swe mice have a later onset of plaque pathology starting at 10–12 months of age and then increasing with age^42^. Both males and females were used and littermates were used as control animals (WT). As further controls, tg-α-syn mice displaying overexpression of human α-synuclein (protein involved in Parkinson's disease)^49^ were used in the PET experiments. The animals were housed with free access to food and water in rooms with controlled temperature and humidity in an animal facility at Uppsala University.

### Radiochemistry

Direct radioiodination of the four proteins F(ab′)_2_-h158, 8D3, 8D3-F(ab′)_2_-h158 and 8D3-F(ab′)_2_-Synagis with iodine-125 (^125^I) for *ex vivo* experiments and iodine-124 (^124^I) for PET experiments was performed using Chloramine-T (ref. 50). The method is based on electrophilic attack of the phenolic ring of tyrosine residues by *in situ* oxidized iodine. Briefly, for ^125^I labelling, 250 pmoles of antibody/fragment or 65 pmoles of fusion proteins (assumed Mw (molecular weight) 270 kDa), ^125^I stock solution (PerkinElmer Inc., Waltham, MA, USA) and 5 μg Chloramine-T (Sigma Aldrich, Stockholm, Sweden) were mixed in PBS to a final volume of 110 μl. The reaction was allowed to proceed for 90 s and subsequently quenched by addition of double molar excess of sodium metabisulfite (Sigma Aldrich) and dilution to 500 μl in PBS. Fab-8D3 had to be modified with Bolton Hunters reagent (Sulfo-SHPP)^51^ (Pierce) before radioiodination to introduce extra phenolic rings for the iodine to target. Fab-8D3 (1 mg ml^−1^ in PBS) was incubated for 30 min with 100 × molar excess of sulfo-SHPP (50 mM in H_2_O) and purified from unbound sulfo-SHPP with a Zeba mini desalting column, Mw cutoff 7 kDa (Pierce). Modified Fab-8D3 was radiolabelled as above.

For ^124^I labelling, 58 μl ^124^I stock solution (Perkin-Elmer Inc.) was pre-incubated for 10 min with 12 μl 50 μM NaI, before addition of 260 pmoles of fusion proteins and 40 μg Chloramine-T in PBS to a final volume of 450 μl. The reaction was allowed to proceed for 120 s and subsequently quenched by addition of 80 μg of sodium metabisulfite in PBS. The radiolabelled proteins were purified from free iodine and low-molecular weight components with a disposable NAP-5 size exclusion column, Mw cutoff 5 kDa (GE Healthcare AB, Uppsala, Sweden), according to the manufacturer's instructions, and eluted in 1 ml of PBS. The yield was calculated based on the added radioactivity and the radioactivity in the purified radioligand solution. Labelling was always performed <2 h before each study. Affinity for Aβ protofibrils and/or TfR was tested with ELISA on the same day as the labelling and the start of the study.

[^11^C]PIB was synthesized as previously described^1^.

Injected radioactivity, specific activity, labelling reaction yields for F(ab′)_2_-h158, 8D3, Fab-8D3, 8D3-F(ab′)_2_-h158, 8D3-F(ab')_2_-Synagis and [^11^C]PIB are given in Table 2.

Deiodination of [^124^I]8D3-F(ab′)_2_-h158 was assessed in the injection solution before the experiment and in plasma samples from injected animals after PET experiments, by separation with Zeba mini desalting columns, where free iodine was trapped in the column.

### *Ex vivo* studies

Mice were anaesthetized with isoflurane at 2, 4, 24 or 72 h after a single i.p. injection of [^125^I]F(ab′)_2_-h158, [^125^I]8D3, [^125^I]Fab-8D3, [^125^I]8D3-F(ab′)_2_-h158 or 8D3-F(ab′)_2_-Synagis. A blood sample was obtained from the heart followed by intracardiac perfusion with 50 ml physiological saline during 2 min. Following perfusion, brains were isolated and the left hemisphere immediately frozen. The right hemisphere was either immediately frozen (for *ex vivo* autoradiography) or left to incubate in 4% paraformaldehyde for 24 h, before it was immersed in a sucrose gradient (10, 20 and 30%) in PBS for cryoprotection (for immunohistochemistry) before being sectioned on a cryostat at −20 °C.

In addition to the terminal blood samples obtained in all animals, blood samples (8 μl) were obtained from the tail vein for a subset of animals also at 0.5, 1, 2, 3, 4, 6, 8, 24, 48 and 72 h after injection.

Radioactivity in blood samples and in the frozen brain hemisphere was measured with a γ-counter (1480 Wizard, Wallac Oy, Turku, Finland). The brain and blood concentrations, quantified as % ID per g tissue, were calculated as follows:

% ID per g=measured radioactivity per gram brain tissue (or blood)/injected radioactivity.

In addition, the brain-to-blood (*K*_p_) concentration ratio was calculated as follows:

*K*_p_=measured radioactivity per gram brain tissue/measured radioactivity per gram blood.

The number of transgenic and WT animals included in the *ex vivo* and PET studies and injected radioactivities of the different radioligands are given in Table 2. Values for % ID per g and *K*_p_ are summarized in Table 1.

### Positron emission tomography

*In vivo* brain distribution of [^124^I]8D3-F(ab′)_2_-h158 in tg-ArcSwe (*n*=13), tg-Swe (*n*=4), WT (*n*=13) and tg-α-syn (*n*=4) mice and of [^124^I]8D3-F(ab′)_2_-Synagis in tg-ArcSwe (*n*=2) and WT (*n*=1) was visualized with PET during 60 min 72 h post i.p. administration of respective radioligand. The day before injection of ^124^I-labelled fusion protein, animals were given water supplemented with 0.2% NaI to reduce thyroidal uptake of ^124^I. A subset of the animals, tg-ArcSwe (*n*=6), tg-Swe (*n*=4) and WT (*n*=4), were investigated with PET during 20 min, starting 40 min after intravenous administration of [^11^C]PIB, 1 week before the [^124^I]8D3-F(ab′)_2_-h158 scans.

At every scanning occasion, the animal was placed in the gantry of the animal PET/computed tomography (CT) scanner (Triumph Trimodality System, TriFoil Imaging, Inc., Northridge, CA, USA) and scanned in list mode followed by a CT examination for 3 min (field of view=8.0 cm). Mice were scanned in a random order.

The PET data were reconstructed using a maximum likelihood expectation maximization (MLEM) two-dimensional algorithm (10 iterations). The CT raw files were reconstructed using filter back projection. All subsequent processing of the PET and CT images were performed in imaging software Amide 1.0.4 (ref. 52). The CT scan was manually aligned with a T2-weighted, magnetic resonance imaging-based mouse brain atlas^53^ containing outlined regions of interests for hippocampus, striatum, thalamus, cortex and cerebellum. The PET image was then aligned with the CT, and thus, the magnetic resonance imaging atlas was also aligned with the PET data. The PET data shown in Figs 5a,c,d and 7a–c are summed images, that is, representing the average activity during the whole PET scan. The PET data were quantified as a concentration ratio of the radioactivity in five regions of interest (whole brain, cortex, hippocampus, thalamus and striatum) to that in cerebellum.

### Biochemical and histopathological analyses

Brain concentrations of soluble Aβ and total Aβ were measured as described previously^18^. In short, mice were saline perfused and the left hemisphere of each animal was homogenized at a 1:5 weight:volume ratio in TBS with complete protease inhibitors (Roche), using a tissue grinder with Teflon pestle (2 × 10 strokes on ice). A volume of 400 μl of each sample was mixed with 400 μl TBS and centrifuged for 1 h at 16,000*g* to obtain a preparation of soluble proteins. The supernatants were aliquoted and stored at −80 °C until analysis. To obtain a preparation including insoluble proteins found in amyloid plaques, 270 μl of the original TBS extract was mixed with 730 μl of concentrated formic acid, to a final formic acid concentration of 70%, followed by homogenization and centrifugation as above.

In the Aβ protofibril ELISA, 96-well plates were coated overnight with 200 ng per well mAb158, and blocked with 1% BSA in PBS. TBS extracts were diluted 1:25 and incubated overnight at +4 °C, followed by detection with biotinylated mAb158 (0.5 μg ml^−1^) and streptavidin-HRP (1:2,000; Mabtech AB). Signals were developed with K blue aqueous TMB substrate (Neogen Corp., Lexington, KY, USA) and read with a spectrophotometer at 450 nm.

For ELISA measurement of Aβx-40 and Aβx-42, 96-well plates were coated overnight with 100 ng per well of polyclonal rabbit anti-Aβ40 or anti-Aβ42 (Agrisera, Umeå, Sweden), and blocked with 1% BSA in PBS. Formic acid extracts were neutralized with 2 M Tris and diluted 500–50,000 times depending on Aβ content and incubated overnight at +4 °C, followed by detection with biotinylated mAb1C3 (0.5 μg ml^−1^)^25^,^26^ and streptavidin-HRP (1:5,000; Mabtech AB). Signals were developed and read as above. All sample and secondary antibody dilutions were made in ELISA incubation buffer (PBS with 0.1% BSA, 0.05% Tween and 0.15% Kathon).

CD31 immunohistochemistry and nuclear track emulsion autoradiography were performed on semi-adjacent sections (20 μm) of the right hemisphere, immunostained for the endothelial marker CD31. First, sections were washed in PBS and incubated with 3% H_2_O_2_ and 10% methanol in water for 15 min. Nonspecific binding was then blocked using 3% BSA in PBS-Tween (0.1%) for 1 h, followed by an overnight incubation with 0.5 μg ml^−1^ rat anti-mouse CD31 (550274, BD Biosciences, San Jose, CA, USA) at 4 °C. The sections were then incubated with 5 μg ml^−1^ biotinylated goat anti-rat (BA-9400, Vector Laboratories Inc., Burlingame, CA) for 1 h at room temperature, followed by PBS washes and a 45-min incubation with avidin/biotin complex (Vector Laboratories). The staining was then visualized with a 3-min 3,3′-diaminbenzidine (DAB) development, for some sections followed by immersion in Congo red for 45 min. Sections were then dehydrated in ethanol (70, 95 and 100%) and left to air dry.

Immediately after CD31 and Congo staining, sections were immersed for 3 s in Ilford K5 emulsion (40 °C), left to air dry for 2 h and stored at +4 °C protected from light. Sections were then developed with Ilford photographic reagents, briefly counterstained with haematoxylin, dehydrated with ethanol and xylene, and mounted with DPX mounting medium.

### *Ex vivo* autoradiography

After PET imaging, a subset of the [^124^I]8D3-F(ab′)_2_-h158-injected mice and the [^124^I]8D3-F(ab′)_2_-Synagis-injected mice was selected for *ex vivo* autoradiography directly after PET imaging. Following saline perfusion, the right hemisphere was instantly frozen and cryosectioned (20 μm). Two sections from each animal and ^124^I-abelled standards of known radioactivity were placed in an X-ray cassette and exposed to positron-sensitive phosphor screens (MS, MultiSensitive, PerkinElmer, Downers grove, IL, USA) for 4 days. The plates were scanned in a Cyclone Plus Imager system (Perkin Elmer) at a resolution of 600 dots per inch. The resulting digital images were normalized to the standards and converted to a false colour scale (Royal) with ImageJ for comparison with PET images.

### Statistics

Results reported are presented as mean±s.d. Data were analysed with two-way analysis of variance followed by Bonferroni's *post hoc* test. In cases where the variance was different in the different groups (variance was in general larger in tg-ArcSwe mice than in WT mice), data were log-transformed before analysis to adhere with the analysis of variance assumption of equal variance. Pearson's correlation was used to analyse correlation of brain uptake of radioligands and Aβ protofibril concentrations.

### Study approval

All procedures described in this paper were approved by the Uppsala County Animal Ethics Board (#C216/11, #C110/11 and #C17/14), following the rules and regulations of the Swedish Animal Welfare Agency, and were in compliance with the European Communities Council Directive of 22 September 2010 (2010/63/EU). All efforts were made to minimize animal suffering and to reduce the number of animals used.

## Additional information

**How to cite this article**: Sehlin, D. *et al.* Antibody-based PET imaging of amyloid beta in mouse models of Alzheimer's disease. *Nat. Commun.* 7:10759 doi: 10.1038/ncomms10759 (2016).

## Figures and Tables

**Table 1:** *Ex vivo* brain concentration of [^125^I]8D3-F(ab′)_2_-h158 at 72 h post injection.

	**% Injected activity dose per gram brain tissue**		***K***_**p**_ **(brain-to-blood concentration ratio)**	
**Age (months)**	**Tg-ArcSwe**	**WT**	**Tg-ArcSwe**	**WT**
4	0.03±0.01	0.02±0.01	0.10±0.01	0.09±0.02
12	0.07±0.03***	0.02±0.00	0.28±0.08***	0.09±0.02
>18	0.14±0.04***	0.02±0.00	0.44±0.10***	0.06±0.01

All values are means±s.d. ***P<0.001 comparison between tg-ArcSwe and WT mice by two-way analysis of variance followed by Bonferroni's *post hoc* test.

**Table 2:** Radioligand concentrations and injected doses.

**Radioligand**	**Labelling reaction yield (%)**	**Specific activity (MBq nmol**^−1^**)**	**Injected radioactivity (MBq)**	**Number of studied mice (tg/WT)**
[^125^I]F(ab′)_2_	78±10	130±36	4.9±1.3	3/3
[^125^I]8D3	54±12	55±10	2.4±1.1	4/7
[^125^I]Fab-8D3	55±5.4	25±4.8	4.6±0.1	2/4
[^125^I]8D3-F(ab′)_2_-h158	70±12	136±24	1.4±0.8	29/26
[^124^I]8D3-F(ab′)_2_-h158	74±3.7	218±29	14.5±1.6	21*/13
[^125^I]8D3-F(ab′)_2_-Synagis	43	116	0.8±0.2	2/4
[^124^I]8D3-F(ab′)_2_-Synagis	62	193	13.3±0.3	2/1
	**Radio-chemical purity (%)**	**Specific Activity (MBq nmol**^**−1**^)	**Injected radioactivity (MBq)**	**Number of studied mice (tg/WT)**
[^11^C]PIB	>95	13±18	8.5±4.4	10/4

tg, transgenic; WT, wild type.

Values are means±s.d.

^*^Including (Thy-1)-h[A30P] α-synuclein transgenic mice (*n*=4).
